# Latanoprostene bunod ophthalmic solution 0.024%: a new treatment option for open‐angle glaucoma and ocular hypertension

**DOI:** 10.1111/cxo.12853

**Published:** 2019-01-07

**Authors:** Murray Fingeret, Ian B Gaddie, Marc Bloomenstein

**Affiliations:** ^1^ VA New York Harbor Health Care System, Brooklyn and St. Albans Campus Queens New York USA; ^2^ Gaddie Eye Centers Louisville Kentucky USA; ^3^ Schwartz Laser Eye Center Scottsdale Arizona USA

**Keywords:** intraocular pressure, latanoprost, latanoprostene bunod, nitric oxide, ocular hypertension, open‐angle glaucoma

## Abstract

Latanoprostene bunod (LBN) ophthalmic solution 0.024% is a novel, once‐daily, nitric oxide‐donating prostaglandin analogue for the lowering of intraocular pressure (IOP) in patients with open‐angle glaucoma and ocular hypertension. The IOP‐lowering actions of LBN are mediated by dual mechanisms of the molecule for increasing aqueous humour outflow. The prostaglandin analogue moiety (latanoprost acid) increases uveoscleral outflow, whereas nitric oxide, released by the nitric oxide‐donating moiety (butanediol mononitrate), increases outflow through the trabecular meshwork and the Schlemm's canal. The clinical efficacy and safety of LBN 0.024% in patients with open‐angle glaucoma or ocular hypertension were established in two similarly designed, double‐masked, pivotal phase 3 studies, APOLLO and LUNAR, the pooled three‐month efficacy phase of which demonstrated significantly greater IOP‐lowering of once‐daily LBN 0.024% over twice‐daily timolol 0.5% at all time points. Additional support for the IOP‐lowering effects of LBN 0.024% was provided by two phase 2 studies in patients with open‐angle glaucoma or ocular hypertension (a dose ranging study versus latanoprost and a 24‐hour IOP crossover study versus timolol) and a phase 1 study of healthy volunteers with IOP in the normal range. In addition, long‐term efficacy and safety were demonstrated in the open‐label safety‐extension phases of the phase 3 pivotal studies and a phase 3 52‐week open‐label study of patients with open‐angle glaucoma (including normal‐tension glaucoma) or ocular hypertension. In conclusion, LBN 0.024% has demonstrated both short‐term and long‐term IOP‐lowering efficacy in patients with open‐angle glaucoma or ocular hypertension, including in healthy volunteers and patients with IOP in the normal range, without apparent clinically‐limiting safety or tolerability concerns.

Glaucoma is the third leading cause of irreversible blindness worldwide.^1^ The most common form of this chronic, progressive optic neuropathy is primary open‐angle glaucoma, which was estimated to affect 2.7 million patients in the United States in 2011.^2^, ^3^ Worldwide prevalence is estimated to be approximately 3.5 per cent among populations 40–80 years of age.^4^ While typically characterised by elevated intraocular pressure (IOP), open‐angle glaucoma can develop in the presence of IOP measurements within ranges typically considered normal (normal‐tension glaucoma); in fact, 30 to 92 per cent of glaucoma patients have been reported to have normal IOP, depending on the patient cohort.^5^, ^6^, ^7^

Data from various global regions, including developed countries, suggest that open‐angle glaucoma is undiagnosed in at least 50 per cent of affected individuals, with much higher rates reported in some studies.^8^, ^9^, ^10^, ^11^, ^12^ Further, open‐angle glaucoma can negatively impact the quality of life of patients, even at early stages; with progressive visual field loss, the disease can impair activities of daily living and is associated with a mounting psychological burden on patients and their care‐givers.^13^

Ocular hypertension, or elevated IOP, is an important risk factor for glaucoma, and currently the only one that is modifiable.^14^, ^15^, ^16^, ^17^ The pathology underlying ocular hypertension involves dysfunction of the trabecular meshwork leading to restricted aqueous humour outflow.^18^, ^19^, ^20^ Additional factors associated with an increased risk of glaucoma development or progression include low blood pressure and low ocular perfusion pressure, particularly in patients with normal‐tension glaucoma.^21^, ^22^, ^23^, ^24^

Multiple landmark studies have demonstrated that lowering IOP slows disease progression in patients with advanced or early‐stage glaucoma, and also prevents glaucoma development in patients with ocular hypertension.^25^, ^26^, ^27^, ^28^, ^29^, ^30^, ^31^ Lowering IOP has additionally been shown to slow disease progression in patients with normal‐tension glaucoma.^29^, ^32^, ^33^, ^34^, ^35^ While target IOP should be individualised, IOP consistently < 18 mmHg and/or lowering IOP by 25 to 30 per cent have been associated with less disease progression.^14^, ^16^, ^26^ Of note, every 1 mmHg of IOP‐lowering is associated with an estimated 10 to 19 per cent reduction in the risk of visual field loss progression in patients with open‐angle glaucoma.^28^, ^36^

Medical (pharmaceutical) treatment is a common intervention for IOP lowering in open‐angle glaucoma, and of the options available prostaglandin analogues are most often prescribed as initial medical therapy based on their established efficacy, overall safety/tolerability and convenient dosing.^16^ If there are contraindications to prostaglandin analogues or other usage barriers (for example, cost, side effects, intolerance), alternative therapies include beta‐blockers, alpha 2 adrenergic agonists, parasympathomimetics, and topical and oral carbonic anhydrase inhibitors.^14^, ^16^ It is common for patients to require multiple pharmacologic agents to maintain adequate IOP control.^31^, ^37^, ^38^

Latanoprostene bunod (LBN) ophthalmic solution, 0.024% (Bausch & Lomb Incorporated, Rochester, New York, USA) is a novel nitric oxide‐donating prostaglandin F2α analogue which offers a new effective treatment alternative for lowering IOP. This article reviews the evidence for LBN 0.024% as a treatment option for lowering IOP in patients with open‐angle glaucoma and/or ocular hypertension.

## Latanoprostene bunod dual mechanism of action

On topical ocular instillation, LBN is rapidly metabolised via carboxyl ester hydrolysis into a prostaglandin F (FP) receptor agonist (latanoprost acid, the active component of latanoprost) and a nitric oxide‐donating moiety (butanediol mononitrate).^39^, ^40^ Butanediol mononitrate subsequently releases nitric oxide (active component) and the inactive metabolite 1,4 butanediol.^41^

**Figure 1:**
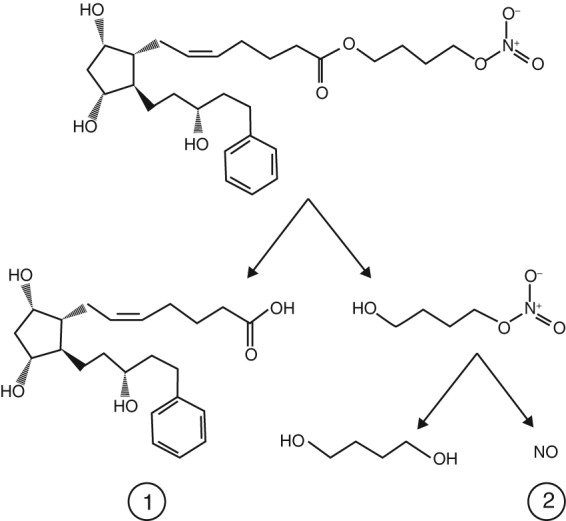
Molecular structure of latanoprostene bunod (LBN) and active metabolites: (1) latanoprost acid and (2) nitric oxide. Reproduced with permission from Taylor & Francis Ltd: Kaufman PL. Latanoprostene bunod ophthalmic solution 0.024% for IOP lowering in glaucoma and ocular hypertension. *Expert Opinion on Pharmacotherapy*, 2017.^42^

**Figure 2:**
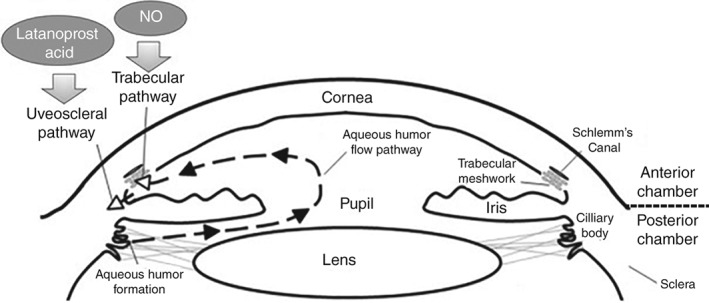
Intraocular pressure‐lowering pharmacologic activity of the active components of latanoprostene bunod. Latanoprost acid increases uveoscleral outflow through extracellular matrix remodelling of the ciliary muscle (uveoscleral/unconventional pathway), whereas nitric oxide increases outflow through relaxation of the trabecular meshwork and the Schlemm's canal (trabecular/conventional pathway). Adapted from Ito and Walter 2013.^44^

The molecular structure of LBN and its active metabolites, latanoprost acid and nitric oxide, are presented in Figure 1.^42^ As a result of these active metabolites, LBN has a dual mechanism of action affecting two distinct pathways for drainage of aqueous humour (Figure 2).^42^, ^43^, ^44^ Latanoprost acid binds to the FP receptor in the ciliary muscle and lowers IOP through extracellular matrix remodelling, thus increasing aqueous humour outflow through the unconventional (uveoscleral) pathway.^45^, ^46^, ^47^ In contrast, nitric oxide lowers IOP by increasing aqueous humour outflow through the primary outflow site, the conventional pathway, through actions on the trabecular meshwork and the Schlemm's canal.^43^, ^48^, ^49^, ^50^, ^51^, ^52^ These mechanisms of action are presumably additive, although the precise contribution of each pathway has not as yet been discerned.

Endogenous nitric oxide is generated by nitric oxide synthases throughout the body and is well known as a regulator of blood flow through relaxation of the vascular smooth muscle.^53^ In the eye it has been shown to play an important physiologic role in IOP regulation by relaxing the cells of the conventional outflow pathway to facilitate trabecular meshwork/Schlemm's canal outflow.^43^, ^54^ A related finding is that nitric oxide markers have been found to be reduced in patients with open‐angle glaucoma, raising the likely possibility that nitric oxide signalling pathways are compromised in these patients.^55^, ^56^, ^57^ Animal data suggest that the effects of nitric oxide on the rate of aqueous humour outflow and IOP are mediated by the enzyme guanylate cyclase‐1 with subsequent activation of the cyclic guanosine monophosphate (cGMP)/protein kinase G signalling cascade.^43^, ^54^, ^58^ An exogenous nitric oxide donor like LBN offers a unique mechanism, targeting the conventional pathway by activating the nitric oxide‐guanylate cyclase‐1‐cGMP cascade, resulting in trabecular meshwork relaxation and thus increased aqueous humour outflow.^43^, ^59^, ^60^

Based on studies with other nitric oxide‐donating compounds, nitric oxide released from LBN may have additional effects on ocular function relevant to glaucoma – namely on ocular blood flow due to its function as a vasodilator and on the optic nerve. With regard to the latter, nitric oxide has been shown to have either neuroprotective or neurodegenerative effects on retinal ganglion cells, depending on the nitric oxide concentration, nitric oxide source and experimental model.^48^, ^61^, ^62^, ^63^

From a safety perspective, a potential concern with nitric oxide is direct oxidative damage to the retinal ganglion cells, reported in association with very high concentrations of this molecule generated by inducible nitric oxide synthase in some animal models.^48^, ^64^ However, due to its short half‐life (estimated at < 3 seconds in extravascular tissues^65^), it is highly unlikely that nitric oxide released from LBN following once daily topical instillation would reach the retina at neurotoxic levels.

The *in vivo* effects of LBN's dual mechanism of action on IOP were studied extensively in preclinical animal models; these data have been recently reviewed in detail^43^ and are summarised briefly here. LBN lowered IOP effectively in several ocular hypertensive glaucoma models (transiently ocular hypertensive rabbits, dogs with inherited glaucoma and primates with laser‐induced ocular hypertension).^43^, ^66^ Of note, in each of the animal models, the IOP lowering with LBN exceeded that of the equimolar concentration of latanoprost alone, likely due to the additive effects of nitric oxide and latanoprost acid when released by LBN into the ocular compartments.^66^

As further evidence supporting an independent IOP‐lowering activity of the nitric oxide moiety, LBN administration reduced IOP in FP receptor knock‐out mice (a model insensitive to the actions of prostaglandin F2α analogues).^67^ In addition, LBN increased cGMP levels and demonstrated a significantly greater relaxation effect on endothelin‐1 contracted human trabecular meshwork cells compared with latanoprost.^68^ Additional IOP lowering over latanoprost was apparent for up to six hours in the animal models (the longest time point evaluated) following topical administration of LBN.^66^ This suggests that although the half‐life of nitric oxide is brief, the sequential metabolism of LBN and resulting signalling cascade activated by release of nitric oxide result in a sustained IOP lowering effect.^43^, ^69^

Taken together, these preclinical studies in multiple animal models of glaucoma/ocular hypertension and in trabecular meshwork cells *in vitro* provided support for the evaluation of LBN in humans.

## LBN 0.024%: clinical trials overview

The clinical efficacy and safety of LBN 0.024% in patients with open‐angle glaucoma or ocular hypertension were established in two similarly‐designed, double‐masked, pivotal phase 3 studies, both with an open‐label safety extension phase.^70^, ^71^, ^72^ Additional supportive studies included a one‐year open label phase 3 study,^41^ a phase 2 dose‐ranging study versus latanoprost 0.005%,^40^ a 24‐hour IOP‐lowering phase 2 study versus timolol maleate 0.5%^73^ and a 24‐hour IOP‐lowering phase 1 study in healthy subjects.^74^

### Phase 1 study

The KRONUS study (NCT01895985) was a phase 1, single‐centre, open‐label study that assessed the IOP‐lowering effects of LBN 0.024% once daily in the evening in 24 healthy Japanese male volunteers (mean age 26.8 years, and mean 24‐hour baseline IOP < 14 mmHg).^74^ Significant reductions from baseline in IOP were observed in the 24‐hour IOP profile after 14 days of LBN 0.024% treatment (p < 0.001); the mean (standard deviation) 24‐hour IOP was 10.0 (1.0) mmHg after 14 days of treatment, corresponding to a 27 per cent reduction from baseline in the study eye (mean [standard deviation] reduction of 3.6 [0.8] mmHg). The findings support a potential IOP‐lowering benefit of LBN 0.024% in open‐angle glaucoma patients even if IOP is within a normal range. Most subjects (22/24) experienced mild ocular adverse events, most commonly conjunctival hyperaemia and punctate keratitis. There were no serious adverse events.

### Phase 2 studies in open‐angle glaucoma and ocular hypertension

The VOYAGER study (NCT01223378) was a phase 2, randomised, investigator‐masked, parallel‐group, dose‐ranging study conducted in the United States and Europe that compared four dosage strengths of LBN (0.006% [n = 82], 0.012% [n = 85], 0.024% [n = 83] and 0.040% [n = 81]) with latanoprost 0.005% (Xalatan; n = 82), each administered once daily in the evening, in patients with open‐angle glaucoma (including pigmentary or pseudoexfoliative glaucoma) or ocular hypertension.^40^

Subjects had to have a baseline IOP of ≥ 24 mmHg for at least two of three baseline measurements (8:00, 12:00, 16:00 hours) in the study eye. Significant reductions from baseline in mean diurnal IOP were observed in all treatment groups at all follow‐up visits (p < 0.0001); dose‐dependent IOP reductions were observed in the LBN groups with an apparent plateau in the dose range of 0.024–0.040%. For the primary endpoint, change from baseline in diurnal IOP at day 28, significantly greater reductions were achieved in the LBN 0.024% and 0.040% groups compared with the latanoprost group (both comparisons, p ≤ 0.01).

The difference between the LBN 0.024% group and the latanoprost group was 1.23 mmHg. In addition, the proportion of subjects with mean diurnal IOP ≤ 18 mmHg was significantly greater in the LBN 0.024% group at all visits (p ≤ 0.05) compared with the latanoprost group, even at day 29 (36–44 hours after the last dose).^40^ The number of subjects with ≥ 1 ocular adverse event was numerically higher in the LBN groups compared with the latanoprost group, with instillation site pain being most commonly reported. Ocular adverse events were generally transient and mild or moderate in severity. Hyperaemia was commonly reported and similar across the treatments.

The CONSTELLATION study (NCT01707381) was a randomised crossover study conducted in the United States in patients with open‐angle glaucoma or ocular hypertension (n = 25).^73^ The study compared the diurnal and nocturnal effects of LBN 0.024% once daily in the evening with timolol maleate 0.5% twice daily on IOP and ocular perfusion pressure. Sitting and supine position ocular perfusion pressures were calculated, using formulas based on the mean arterial blood pressure and IOP, adjusted for the height of the eye over the heart. At baseline, after four weeks of initial treatment, and after four weeks of crossover treatment, 24‐hour IOP and blood pressure measurements were obtained in a sleep laboratory (every two hours in sitting and supine positions during the 16‐hour diurnal/wake period and in the supine position during the eight‐hour nocturnal/sleep period).

During the diurnal/wake periods (while sitting and supine) both LBN and timolol significantly lowered IOP from baseline (all p < 0.001) with no difference between the treatment groups; however, during the nocturnal/sleep period, the supine IOP was significantly lowered from baseline with LBN 0.024% treatment (p = 0.002), but not with timolol 0.5%, and a significant difference between treatment groups was observed (p = 0.004). LBN 0.024% was associated with greater diurnal sitting and supine ocular perfusion pressure compared with baseline (p ≤ 0.006) and greater nocturnal supine ocular perfusion pressure compared with timolol treatment (p = 0.010). Neither treatment impacted mean arterial blood pressure in this study, limiting its impact on ocular perfusion pressure calculations. There were two adverse events recorded for LBN 0.024% (punctate keratitis and instillation site erythema) and four for timolol (three punctate keratitis and one instillation site irritation).

### Phase 3 studies in open‐angle glaucoma and ocular hypertension

Phase 3 studies of LBN 0.024% for the treatment of open‐angle glaucoma and ocular hypertension included the similarly designed APOLLO^70^ and LUNAR^71^ studies, which both included a three‐month double‐masked efficacy phase followed by a three‐month (LUNAR) or nine‐month (APOLLO) open‐label safety extension phase and the one‐year open‐label JUPITER^41^ study (Table 1).

**Table 1:** Latanoprostene bunod ophthalmic solution, 0.024%: summary of phase 3 clinical studies in patients with ocular hypertension or open‐angle glaucoma

Study	Study treatment(s); (number of subjects) and study duration	Key efficacy finding(s)	Ocular adverse events
APOLLO Randomised, controlled, double‐masked study^70^ followed by safety extension	Double‐masked efficacy phase: LBN 0.024% once daily in the evening (n = 284) Timolol 0.5% twice daily (n = 133) Duration of double‐masked phase: three months	Double‐masked efficacy phase: Mean IOP significantly lower with LBN 0.024% vs timolol 0.5% (p ≤ 0.002, at all nine efficacy time points),† demonstrating both non‐inferiority and superiority of LBN 0.024% vs timolol 0.5%	Double‐masked efficacy phase: Proportion of eyes with ≥ 1 ocular adverse event was comparable between groups Adverse events reported in ≥ 1% of eyes in both treatment groups included eye irritation, conjunctival hyperaemia, eye pain, dry eye and instillation site pain Most were mild–moderate in severity
LUNAR Randomised, controlled, double‐masked study^71^ followed by safety extension	Double‐masked efficacy phase: LBN 0.024% once daily in the evening (n = 278) Timolol 0.5% twice daily (n = 136) Duration of double‐masked phase: three months	Double‐masked efficacy phase: Mean IOP significantly lower with LBN 0.024% vs timolol 0.5% (p ≤ 0.025, at the majority of efficacy time points),† ^,^ ‡ demonstrating non‐inferiority of LBN 0.024% vs timolol 0.5%	Double‐masked efficacy phase: Proportion of patients with ≥ 1 ocular adverse events appeared greater for the LBN 0.024% group vs timolol 0.5% group Adverse events reported in ≥ 1% of study eyes in both treatment groups included eye irritation, eye pain and blurry vision Conjunctival and ocular hyperaemia were reported in more patients in the LBN 0.024% group (9% and 2.5%, respectively) than in the timolol group (both < 1% of patients) Most were mild–moderate in severity
Open‐label safety extensions of APOLLO and LUNAR studies (pooled analysis)^72^	Pooled open‐label safety extension: LBN 0.024% once daily in the evening (n = 769) Duration of safety extensions: APOLLO: nine months LUNAR: three months	Pooled open‐label safety extension: Patients in the LBN 0.024% group during the double‐masked efficacy phase maintained consistently lowered IOP during the open‐label extension phase, with a mean (standard deviation) diurnal IOP of 18.1 (2.9), 18.2 (3.3) and 17.9 (3.0) mmHg at months six, nine and 12,§ respectively, of the open‐label extension phase, compared to 18.1 (2.9) at month three of the double‐masked phase Patients treated with timolol during the double‐masked efficacy phases had an additional and sustained decrease in mean diurnal IOP when crossed over to LBN 0.024% in the open‐label extension study phases	Pooled double‐masked plus open‐label safety extension: Most common ocular adverse events were conjunctival hyperaemia (5.9%), eye irritation (4.6%) and eye pain (3.6%) The majority (≥ 97%) were mild–moderate in severity
JUPITER Single‐arm,open‐label study^41^	LBN 0.024% once daily in the evening (n = 130) 52 weeks	At week 52, IOP reduction from baseline was 26.3% and 23.0% in study eyes and treated fellow eyes, respectively (both p < 0.001) Significant IOP reduction from baseline in both eyes starting at week four and for all subsequent visits (all p < 0.001)	Most frequently reported ocular adverse events: conjunctival hyperaemia (17.7%), growth of eyelashes (16.2%), eye irritation (11.5%) and eye pain (10.0%) No severe ocular adverse events reported

IOP: intraocular pressure, LBN: latanoprostene bunod.

† Nine efficacy time points were: 8:00, 12:00, 16:00 hours at each post‐baseline visit (week two, week six and month three).

‡ Exception: LBN 0.024% did not meet the criteria for statistical superiority over timolol at the 8:00 hours time point at week two.

§ Total treatment time was 12 months for those patients in the APOLLO study who were initially randomised to three months of LBN 0.024% during the double‐masked phase and had an additional nine months of LBN 0.024% during the open‐label extension phase.

APOLLO (NCT01749904) and LUNAR (NCT01749930) were phase 3, randomised, multicentre, double‐masked, parallel group studies conducted in the United States and Europe which compared LBN 0.024% once daily in the evening (and, for masking purposes, vehicle in the morning) with twice daily timolol 0.5% for three months in patients with open‐angle glaucoma (including pigmentary or pseudoexfoliative glaucoma) or ocular hypertension.^70^, ^71^

In both studies, patients had a mean IOP ≥ 24 mmHg at baseline in the study eye (three measurement points). During the three‐month double‐masked phases of these studies, IOP was measured at nine time points (8:00, 12:00, 16:00 hours at week two, week six and month three). In the APOLLO study, the LBN 0.024% group (n = 284) had significantly lower mean IOP values in the study eye compared with the timolol 0.5% group (n = 133) at all efficacy time points.^70^ Mean IOP values in the LBN 0.024% group ranged from 17.8 to 18.7 mmHg and from 19.1 to 19.8 mmHg in the timolol group (p ≤ 0.002 at all time points).

These findings demonstrated both non‐inferiority and superiority for LBN 0.024% versus timolol 0.5% based on the following criteria: non‐inferiority was confirmed when the upper limit of the 95 per cent confidence interval (CI) for the difference between treatments did not exceed 1.5 mmHg at all nine time points and did not exceed 1 mmHg for five of the nine time points; superiority was demonstrated by the upper limit of the 95 per cent CI not exceeding 0 mmHg at all nine time points.

An IOP ≤ 18 mmHg was achieved at all nine time points in 22.9 per cent of patients in the LBN 0.024% group versus 11.3 per cent in the timolol group (difference 11.6 per cent, 95 per cent CI 4.3–18.9; p = 0.005); an IOP reduction ≥ 25 per cent was achieved at all time points in 34.9 versus 19.5 per cent, respectively (difference 15.3 per cent, 95 per cent CI 6.6–24.0; p = 0.001). Adverse events considered related to study medication were uncommon with both LBN 0.024% (11.0 per cent of study eyes) and timolol (8.9 per cent of study eyes), with most commonly reported events being eye irritation (LBN 3.9 per cent; timolol 2.2 per cent), conjunctival hyperaemia (LBN 2.8 per cent; timolol 1.5 per cent) and eye pain (LBN 1.4 per cent; timolol 2.2 per cent), and most events were mild or moderate in severity.

Conjunctival hyperaemia, prospectively evaluated by investigators at each visit using a photographic reference scale, was observed in approximately 40 per cent of subjects at baseline prior to any treatment. Throughout the three months of double‐masked treatment, the proportion of subjects who had conjunctival hyperaemia only slightly varied from baseline and was comparable between treatment groups; although more patients in the LBN 0.024% had moderate or severe hyperaemia at each study visit (study eyes, LBN 0.024% versus timolol 0.5%: week two, 9.6 versus 0.7 per cent; week six, 11.8 versus 3.8 per cent; month three, 8.5 versus 2.4 per cent).

In the LUNAR study, the mean IOP in the LBN 0.024% group (n = 278) was significantly (p ≤ 0.025) lower as compared with that in the timolol 0.5% group (n = 136) at eight out of nine time points, with the only exception being the first assessment (8:00 hours, week two).^71^ Noninferiority of LBN 0.024% to timolol 0.5% was demonstrated according to the same criteria as in the APOLLO study,^70^ described above. An IOP ≤ 18 mmHg was achieved at all nine efficacy time points in 17.7 per cent of patients in the LBN 0.024% group versus 11.1 per cent in the timolol 0.5% group (difference 6.6 per cent; p = 0.084) and IOP reduction ≥ 25 per cent was achieved at all time points in 31.0 versus 18.5 per cent, respectively (difference 12.5 per cent; p = 0.007).

Both treatments were well‐tolerated, although a numerically higher percentage of patients experienced ≥ 1 ocular adverse event in the study eye in the LBN 0.024% group compared with the timolol 0.5% group (23.8 versus 13.3 per cent, respectively). The most frequently reported ocular adverse events in the LBN 0.024% group were conjunctival hyperaemia (9.0 versus 0.7 per cent with timolol 0.5%), eye irritation (7.2 versus 4.4 per cent with timolol 0.5%) and eye pain (5.8 versus 3.7 per cent with timolol 0.5%). With the exception of one case of severe conjunctival hyperaemia in the LBN 0.024% group, ocular adverse events were mild or moderate in severity.

Investigator‐evaluated conjunctival hyperaemia at baseline was observed in approximately 37 and 41 per cent of patients in the LBN 0.024% and timolol 0.5% groups, respectively.^71^ The proportion of patients assessed by the investigator as having conjunctival hyperaemia was higher in the LBN 0.024% group compared with the timolol 0.5% group (study eyes, week two, 47.8 versus 36.6 per cent; week six, 47.8 versus 34.1 per cent; month three, 48.3 versus 31.5 per cent); moderate to severe conjunctival hyperaemia was observed in approximately six to eight per cent of patients in the LBN 0.024% group compared with one to three per cent of patients in the timolol 0.5% group.^71^

**Figure 3:**
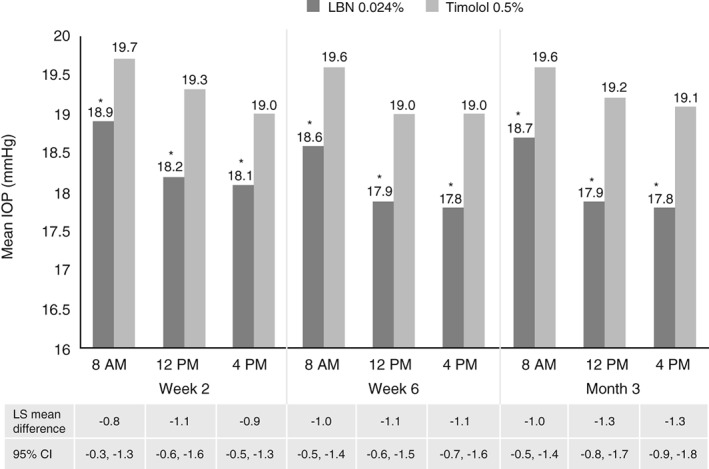
Pooled phase 3 efficacy findings (APOLLO and LUNAR studies), once daily latanoprostene bunod (LBN) 0.024% (n = 562) versus twice daily timolol 0.5% (n = 269). Data represent least squares mean intraocular pressure (IOP) in the study eye by visit and time point by treatment group (intent‐to‐treat population; last observation carried forward).^72^ *p < 0.001. Reproduced with permission from Wolters Kluwer Health – Lippincott Williams & Wilkin: Weinreb RN, Liebmann JM, Martin KR et al. Latanoprostene bunod 0.024% in subjects with open‐angle glaucoma or ocular hypertension: Pooled phase 3 study findings. *Journal of Glaucoma*, 2018; promotional and commercial use of the material in print, digital or mobile device format is prohibited without the permission from the publisher Wolters Kluwer. Please contact http://permissions@lww.com for further information.

The APOLLO and LUNAR study data were pooled, allowing for a robust analysis of data from all 774 subjects who completed the three‐month, double‐masked phases of these studies.^72^ During the double‐masked efficacy phase, mean IOP in the study eye was significantly lower in LBN 0.024%‐treated eyes (range 17.8–18.9 mmHg) than in timolol‐treated eyes (range 19.0–19.7 mmHg) at all nine time points (all p < 0.001; Figure 3).^72^ Across the two studies, the reduction from baseline in IOP ranged from 7.5 to 9.1 mmHg.^42^, ^70^, ^71^ Pooled analysis showed that at three months, the mean percentage reduction in IOP from baseline in LBN 0.024%‐treated subjects was 32 per cent.^72^ The pooled analysis demonstrated both non‐inferiority and superiority of LBN 0.024% over timolol 0.5% for IOP lowering (same criteria as described for individual studies, above).

The three‐month double‐masked phases of the LUNAR and APOLLO studies were followed by open‐label extension phases of three‐ and nine‐months duration, respectively, for total study durations of six months and 12 months, respectively. During the open‐label extension phases, all subjects were treated with once daily LBN 0.024% in the evening (including those treated with timolol during the double‐masked phase); IOP was measured at 8:00, 12:00, 16:00 hours at month six in the LUNAR study and at months six, nine and 12 in the APOLLO study.^72^

**Figure 4:**
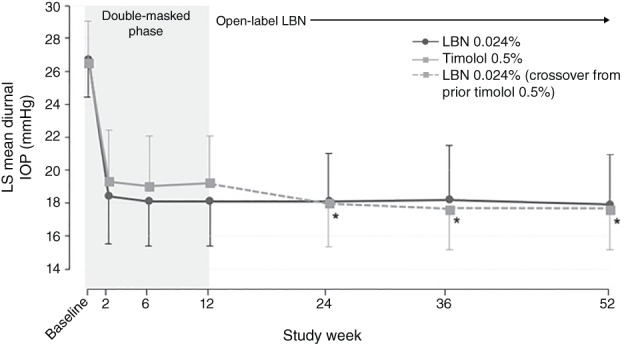
Pooled data from the APOLLO and LUNAR studies, including open‐label extension phases. Mean (standard deviation) diurnal intraocular pressure (IOP) for subjects randomised to latanoprostene bunod (LBN) 0.024% and subjects randomised to timolol in the double‐masked efficacy phase and crossed over to LBN 0.024% in the open‐label safety extension phase (intent‐to‐treat population; data as observed).^72^ *p ≤ 0.009 versus week 12 for subjects randomised to timolol 0.5% in the efficacy phase. Reproduced with permission from Wolters Kluwer Health – Lippincott Williams & Wilkin: Weinreb RN, Liebmann JM, Martin KR et al. Latanoprostene bunod 0.024% in subjects with open‐angle glaucoma or ocular hypertension: Pooled phase 3 study findings. *Journal of Glaucoma*, 2018; promotional and commercial use of the material in print, digital or mobile device format is prohibited without the permission from the publisher Wolters Kluwer. Please contact http://permissions@lww.com for further information.

An integrated analysis of these open‐label extension data found that IOP reduction with LBN 0.024% was maintained through 12 months of treatment, with no apparent loss of IOP‐lowering effect (Figure 4).^72^ Mean diurnal (standard deviation) IOP reductions from baseline with LBN 0.024% treatment were 8.6 (3.0), 8.5 (3.5) and 8.8 (3.2) mmHg at months six, nine and 12, respectively, similar to that noted at month three (8.6 [3.0] mmHg) with LBN 0.024% at the end of the double‐masked efficacy phase.

Subjects who were switched to open‐label LBN 0.024% after receiving timolol 0.5% during the efficacy phase demonstrated an additional 1.1–1.2 mmHg decrease in mean diurnal IOP at six, nine and 12 months (p ≤ 0.009 versus timolol 0.5% at three months; Figure 4). During the entire study (double‐masked and open‐label phases), the overall incidence of adverse events considered related to LBN 0.024% treatment was 17.8 per cent (including data from subjects crossed over to LBN 0.024% during the open label phase) and few subjects (1.4 per cent) discontinued due to ocular adverse events during treatment with LBN.^72^

The most commonly reported ocular adverse events during treatment with LBN 0.024% were conjunctival hyperaemia (5.9 per cent), eye irritation (4.6 per cent) and eye pain (3.6 per cent). Objective assessments of hyperaemia at each study visit found any hyperaemia in 32.6 to 50.0 per cent of study eyes while being treated with LBN 0.024%. Moderate/severe hyperaemia was noted infrequently (3.6–9.7 per cent of LBN‐treated eyes).

### One‐year open‐label study

JUPITER (NCT01895972) was a long‐term, single‐arm, open‐label, multicentre study conducted in Japan that assessed the use of LBN 0.024% once daily in the evening for 52 weeks in 130 patients with open‐angle glaucoma (including normal‐tension glaucoma, pigmentary or pseudoexfoliative glaucoma) or ocular hypertension.^41^ Mean (standard deviation) baseline IOP (measured at 10:00 hours) in study eyes was 19.6 (2.9) mmHg (range 15.0–30.0 mmHg). The majority (74.6 per cent) of baseline IOPs were between 15 and 21 mmHg, which is consistent with an observed phenomenon of normal‐tension glaucoma being common in Japanese populations.^7^, ^41^

**Figure 5:**
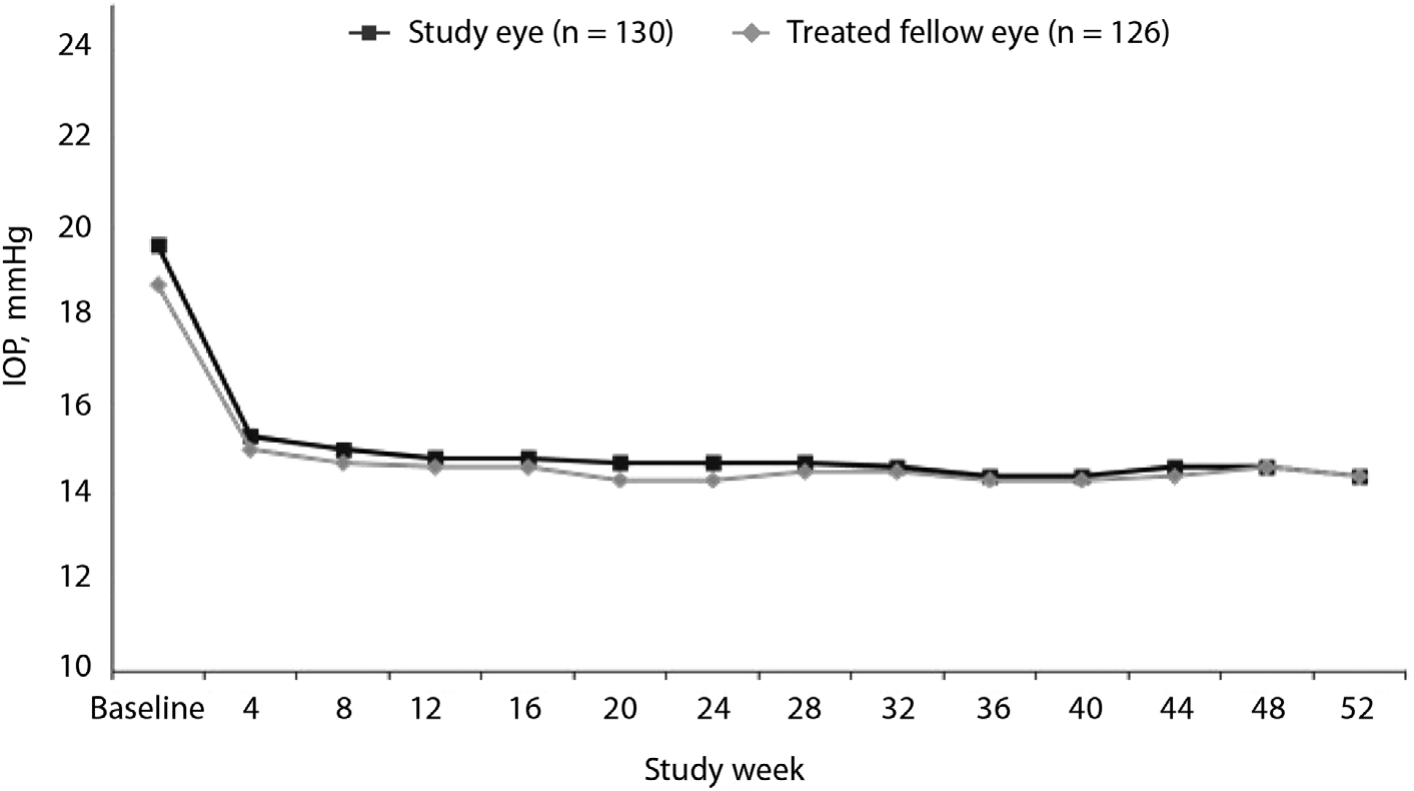
Mean IOP (mmHg) by visit in the JUPITER study, open‐label treatment with LBN 0.024% once daily in Japanese subjects.^41^ All post‐baseline measurements p < 0.001 versus baseline. Standard deviations at each time point ranged from 2.31 to 3.00 mmHg. IOP: intraocular pressure, LBN: latanoprostene bunod. Reprinted by permission from Adis: Adis, part of Springer Science+Business Media, *Advances in Therapy*, Kawase K, Vittitow JL, Weinreb RN et al. Long‐term safety and efficacy of latanoprostene bunod 0.024% in Japanese subjects with open‐angle glaucoma or ocular hypertension: the JUPITER study.

Mean IOP was significantly reduced from baseline by 22.0 per cent (mean [standard deviation] 15.3 [3.0] mmHg) at week four, with even greater decreases observed at all subsequent visits (all time points, p < 0.001). At week 52, mean IOP was 14.4 (2.7) mmHg, a reduction from baseline of 26.3 per cent. Comparable IOP reductions were observed throughout the study in the treated fellow eyes (Figure 5).^41^

This one‐year study confirmed a high level of safety and tolerability with long‐term use of LBN 0.024%; the most common adverse events in study eyes were conjunctival hyperaemia (17.7 per cent), eyelash growth (16.2 per cent), eye irritation (11.5 per cent) and eye pain (10.0 per cent). All adverse events were mild or moderate in severity and no subject discontinued the study because of a treatment‐related adverse event.

Based on investigator assessments, hyperaemia was present in 15.4 per cent of study eyes at baseline and between 17.5 and 20.8 per cent of eyes at each study visit; almost all noted hyperaemia was graded as mild and none were severe. Increased iris pigmentation, as assessed by photography, was noted in nine per cent of LBN 0.024%‐treated eyes, and an additional 14 per cent of eyes were categorised as having a possible increase at one year. By comparison, several studies with latanoprost in Japanese populations have reported investigator‐assessed rates of iris pigmentation > 50 per cent after one year of treatment.^75^, ^76^, ^77^

## Summary and conclusions

LBN is a promising new treatment option for IOP lowering in patients with open‐angle glaucoma and/or ocular hypertension, with pharmacologic activity mediated by the dual actions of a well‐known prostaglandin F2α analogue moiety (latanoprost acid) and a nitric oxide‐donating moiety (butanediol mononitrate). This two‐fold mechanism contributes to improved aqueous humour outflow via the uveoscleral outflow pathway as well as the trabecular meshwork and the Schlemm's canal.

Despite growing evidence that the trabecular meshwork plays a major role in resistance to aqueous humour outflow in open‐angle glaucoma patients, most current IOP‐lowering drugs act on other outflow targets (that is, uveoscleral outflow) or by suppressing aqueous humour production.^15^ Hence LBN, with its action on both the uveoscleral and trabecular meshwork outflow pathways, is a welcome addition to the armamentarium of IOP‐lowering medications.

Research with LBN to date confirms the measurable clinical benefits resulting from the unique dual mechanism of action. Early phase studies established a robust IOP‐lowering effect in both healthy subjects with low baseline IOP^74^ and in individuals with open‐angle glaucoma or ocular hypertension;^40^ in the latter group, the reduction from baseline to day 28 in diurnal IOP with LBN was significantly greater than that observed with latanoprost, considered the current standard of care.^40^ Further, the noted 1.23 mmHg difference between the LBN and latanoprost treatment groups should be seen as clinically relevant given that every 1 mmHg of IOP‐lowering has been associated with an estimated 10–19 per cent reduction in the risk of progression in patients with glaucoma.^28^, ^36^

Once‐daily LBN was also shown to have greater IOP‐lowering efficacy compared with twice‐daily timolol in patients with open‐angle glaucoma or ocular hypertension in several studies,^70^, ^71^, ^73^ including two large phase 3 pivotal trials^70^, ^71^ and a sleep lab study confirming the 24‐hour IOP‐lowering effect of LBN as well as improvement on ocular perfusion pressure over both the diurnal and nocturnal period.^73^

Sustained IOP‐lowering efficacy of LBN over treatment periods up to one year was shown in the open‐label extension phases of the phase 3 pivotal trials in patients with elevated IOP^72^ as well as in a long‐term study in Japanese patients which included patients with normal‐tension glaucoma.^41^

LBN 0.024% was well‐tolerated in these clinical trials, with adverse event findings generally typical of topical prostaglandin analogue therapy. Notably, there were few discontinuations due to ocular adverse events in these studies and no changes in visual acuity or visual fields with repeat dosing as long as one year.^41^, ^72^

A study demonstrating the impact of LBN 0.024% on visual fields preservation, similar to the United Kingdom Glaucoma Treatment Study which demonstrated a significant reduction in visual field deterioration with latanoprost 0.005% compared with placebo,^30^ would be an interesting avenue for future research into the impact of LBN 0.024% on disease progression.

In conclusion, a range of clinical trial experience has established both the short‐term and long‐term efficacy of once daily LBN 0.024% IOP lowering among patients with open‐angle glaucoma or ocular hypertension, including normal‐tension glaucoma, without apparent clinically limiting safety or tolerability concerns. Ongoing real‐world clinical experience with LBN will provide further answers to the role of LBN in the management of patients who require IOP lowering, including sustained benefit over multiple years of use. Such experience will determine whether this unique dual‐action compound can lessen the need for combination therapy; if the once daily dosing regimen translates into patient adherence benefits; and to what degree any or all of these features can improve patient health, lessen the need for surgery and possibly impact overall glaucoma treatment costs.
